# A microsatellite based multiplex PCR method for the detection of chromosomal instability in gastric cancer

**DOI:** 10.1038/s41598-018-30971-z

**Published:** 2018-08-22

**Authors:** Meike Kohlruss, Magdalena Reiche, Moritz Jesinghaus, Bianca Grosser, Julia Slotta-Huspenina, Alexander Hapfelmeier, Lukas Bauer, Alexander Novotny, Wilko Weichert, Gisela Keller

**Affiliations:** 1 Department of Pathology, Technical University of Munich, Trogerstr. 18, 81675 Munich, Germany; 2 Department of Medical Informatics, Statistics and Epidemiology, Technical University of Munich, Ismaningerstr. 22, 81675 Munich, Germany; 3 Department of Surgery, Technical University of Munich, Ismaningerstr. 22, 81675 Munich, Germany; 4 German Cancer Consortium (DKTK), Partner Site Munich, Technical University, Trogerstr. 18, 81675 Munich, Germany

## Abstract

Chromosomal instability (CIN) is a hallmark of distinct subclasses of tumours with potential clinical relevance. The aim of our study was to establish a time and cost effective method for the determination of CIN in gastric carcinomas (GC). We developed a microsatellite based multiplex PCR assay for the detection of allelic imbalances (AI) using experimentally defined marker specific threshold values for AI. The assay was tested in 90 formalin-fixed paraffin-embedded GC and results were compared in a subset of 30 carcinomas with the Affymetrix OncoScan assay, which detects copy number variations on genome wide level. The ratios of alterations detected by the two methods demonstrated a significant correlation (r = 0.88). Based on the results of the OncoScan assay, tumours were classified in CIN-High and CIN-Low and a threshold of the AI ratio determined with the PCR assay was defined. Accordingly, 20 of the 90 GC (22%) were CIN-Low and 70 (78%) CIN-High. A significant association of CIN-High was found with intestinal type tumours and proximal tumour localization. In conclusion, we established a PCR based method to categorize AI as surrogate for CIN, which is easy to perform and useful for the clarification of the clinical relevance of CIN in large GC cohorts.

## Introduction

The development of genomic instability is considered to endow cells with genetic alterations that drive cancer development and progression. Chromosomal instability (CIN) is considered to be a main driving force for the frequently observed aneuploidy of tumour cells. CIN is suggested to describe a rate of losses or gains of whole or parts of chromosomes occurring during an ongoing process^1^. The term CIN however, is not clearly defined and is often used just to quantitatively describe the extent of chromosomal changes observed in a given tumour as a surrogate for CIN. The reasons for CIN may be diverse and are still poorly understood^1,2^.

In the context of The Cancer Genome Atlas (TCGA) project a comprehensive molecular profiling of 295 gastric carcinomas (GC) was performed and four main molecular subgroups were identified: 1) tumours with microsatellite instability (MSI), 2) EBV positive tumours, 3) genomically stable tumours and 4) tumours with CIN^3^. Recently, an algorithm based on protein and mRNA expression has been proposed for a simplified classification of the four subgroups^4^. In addition gene expression based models to predict these subgroups have been demonstrated to successfully stratify the patients by survival and outcome to adjuvant chemotherapy^5^. However the overall clinical relevance of this molecular classification is still poorly characterized. An analysis of various GC cohorts encompassing considerably high number of patients and addressing different clinically relevant questions is necessary to estimate the full potential value of this “omics” based classification system.

CIN in the TCGA study of GC and also of oesophageal adenocarcinomas refers to the description of the extent of chromosomal alterations in the tumours mainly determined by genome-wide single nucleotide polymorphism (SNP) based microarray technology^3,6,7^. This approach, however, is obviously not suited for routine diagnostics since it is relatively cost intensive, requires specific experimental equipment and is preferentially performed on DNA from fresh frozen tumour tissues.

The aim of our study was therefore to establish a relatively simple and cost effective method for the determination of CIN in GC, which should be applicable to DNA isolated from formalin-fixed paraffin-embedded (FFPE) tumour tissues.

Microsatellite analysis has frequently been used to detect loss of heterozygosity (LOH) or allelic imbalance (AI) in tumours in numerous studies and thus we implemented a microsatellite based multiplex PCR assay for the detection of AI^8–12^. We determined individual marker specific cut-off values for the definition of AI essentially as described and analysed a GC cohort for AI^10^. We then asked if the results could be used as a surrogate for CIN by comparing it to a genome-wide analysis of chromosomal alterations in a subset of the tumours. For this purpose we used the Affymetrix OncoScan method, which is a SNP based array technology using molecular inversion probes. Finally we analysed our assay in relation to tumour heterogeneity and to the minimal tumour cell content for reliable CIN detection.

## Material and Methods

### Material

We used 58 non-tumorous FFPE tissues from 11 GC patients to determine individual cut-off values for the definition of AI for each microsatellite marker. The non-tumorous tissues comprised histologically normal mucosa of the stomach and tumour free lymph nodes.

Tissues from 100 primarily resected GC patients who were operated at the Department of Surgery at the Technical University of Munich between 2001 and 2013 were analysed. Selection criteria were no treatment with preoperative chemotherapy and availability of material. Patients’ characteristics are shown in Supplementary Table S1. In a preselection step, all tumours were analysed for MSI. As only stable microsatellite markers allow a clear evaluation of AI only microsatellite stable (MSS) tumours (n = 90) were analysed for AI and CIN.

A subset of 30 tumours was - in addition - analysed for genome wide copy number alterations by the Affymetrix OncoScan assay. The criterion for the selection of the 30 cases for the OncoScan analysis was an approximately balanced proportion of tumours with high and low/median frequencies of AI detected with the microsatellite based multiplex PCR assay.

### Ethic statement

The use of tissue samples was approved by the local Institutional Review Boards at the Technical University Munich (reference: 502/15s). The participants or their legal representatives had given informed consent. All experiments were performed in accordance with relevant guidelines and regulations.

### DNA isolation

DNA from paired tumour and non-tumorous FFPE tissues was isolated after microdissection from 8 µm thick sections after deparaffinization and proteinase K digestions using the Maxwell extraction system according to the instructions of the manufacturer (Promega, Madison, WI) or using a FFPE DNA purification kit (Qiagen, Hilden, Germany). Only samples with a tumour cell content of at least 25%, which corresponded to the limit of detection of the AI ratio, which we had determined for the multiplex PCR assay, were included.

### Analysis for microsatellite instability

MSI was analysed using the five markers BAT25, BAT26, D2S123, D5S346 and D17S250 recommended by the National Cancer Institute^13^ and is described in detail in the Supplementary Methods. MSI was scored positive if at least two of the five markers showed MSI in the tumour^13^.

### Establishing the microsatellite based multiplex PCR assays for the detection of AI

A panel of 30 microsatellite markers covering 14 chromosomal regions, which demonstrated gains or losses of parts or whole chromosomes in GC at various frequencies according to the TCGA data^3^ was initially chosen (Table 1). Selection criteria were the rate of heterozygosity ≧75% and the size of the PCR product between 100–230 bp as indicated^14,15^. The chromosomal positions of the microsatellite loci were reviewed with the NCBI Map Viewer (NCBI, Bethesda, MD). The software Multiplex Manager 1.2 was used to virtually design multiplex PCR reactions^16^. Amplification of 30 markers was performed in 5 multiplex PCRs using the Type-it Microsatellite PCR kit (Qiagen, Hilden, Germany) and the respective forward primers were labelled with the dyes FAM, HEX or ATTO550 (Eurofins Genomics, Ebersberg, Germany). 20 ng DNA was added to each PCR reaction in a final volume of 25 µl. The cycle conditions are described in Supplementary Methods and the final compositions of the five multiplex PCR reactions are summarized in Supplementary Table S2.

**Table 1:** Information about the selected microsatellite markers.

Region	Marker name	Forward primer	Reverse primer	Het^‡^	Size range [bp]
2p21	D2S123^†^	AAA CAG GAT GCC TGC CTT TA	GGA CTT TCC ACC TAT GGG AC	0.76^§^	197–227
4q22.1	D4S423	TTGAGTAGTTCCTGAAGCAGC	CAAAGTCCTCCATCTTGAGTG	0.82	103–125
4q22.1	D4S1534*	ATTCAGTTTCAGCCCCAT	ACCAGCCCAAGGTAGAGG	0.76	146–158
5q11-q12	D5S624	CTATGTAACAAACCTGCATGTTGTG	ATTTGCTGAACGAATGACCC	0.83	146–166
5q11-q12	D5S2107*	AGCCTTTGGGCCAACA	CAAACCAACAGGAGTATGTACTTTT	0.85	166–188
5q21-22	D5S346^†^	ACTCACTCTAGTGATAAATCGGG	AGCAGATAAGACAGTATTACTAGTT	—	96–122
6p25.3	D6S1713*	AATCACTGTTACCCATAGGGTTATC	AGGCCAAGACCTCTGTGC	0.9	134–172
6p25.3	D6S1617	TGCAAAACAGGCACACATAC	TTAATCAATTTTCTGCAAAGATAAA	0.86	101–123
7q21	D7S492	ATCTTGGATTTAGGGTTGGC	GGCTCTGCTCCATCTTCATA	0.76	145–155
7q31	D7S486	AAAGGCCAATGGTATATCCC	GCCCAGGTGATTGATAGTGC	0.8	133–146
8p23.1	D8S552	AGGATTGTAATTTCCTTGC	GGGACTTTTTGAAGGTTTG	0.79	168–182
8p23.1	D8S261*	TGCCACTGTCTTGAAAATCC	TATGGCCCAGCAATGTGTAT	0.78	128–144
8q24.21	D8S1793	TGAGCCGAGTTCTTACCAC	AACAAGTCCAGCTTGATGAG	0.82	113–147
8q24.21	D8S1720	GTGCCACCTGCCTGAA	CCACTACCTATTTAGAGAGGCCA	0.81	130–144
8q24.21	D8S1801*	AGGCTGGGTCCTGATG	TTTCCGTCTGTGATTACAGT	0.81	211–235
9p21	D9S157	AGCAAGGCAAGCCACATTTC	TGGGGATGCCCAGATAACTATATC	0.83	133–149
9p21	D9S171	AGCTAAGTGAACCTCATCTCTGTCT	ACCCTAGCACTGATGGTATAGTCT	0.79	159–177
12p12.1	D12S1682	GGGACAAGAGTGAGACTTGG	CCTTTATTGAAGTAAACTGTGAAGC	0.77	133–151
12p12	D12S1631	TGGGCTCATCTGGAAA	GGAGGCAAACACTGATAACTTAC	0.84	161–185
16q23.1	D16S3125*	TGTCAGGCTCACAGCA	CCGCTACAGCAGCATTT	0.8	183–205
16q23	D16S507	GCAGGGGCTAGAAGGTG	TGTTCGCCTCTTGCAGT	0.79	175–195
17p13.1	D17S1353*	CTGAGGCACGAGAATTGCAC	TACTATTCAGCCCGAGGTGC	0.87	200–222
17p13	D17S796	CAATGGAACCAAATGTGGTC	AGTCCGATAATGCCAGGATG	0.8	144–174
17p13	D17S1832*	ACGCCTTGACATAGTTGC	TGTGTGACTGTTCAGCCTC	0.81	179–195
17q12	D17S946*	ACAGTCTATCAAGCAGAAAAATCCT	TGCCGTGCCAGAGAGA	0.8	128–142
17q12	D17S1872	CCAACTCTAGGACTGGGG	AATTGGGTCCAGAGAGCA	0.89	108–140
17q12	D17S1861	AGGGGCAGCAGTCCTGTA	ACATCATCCTGAAATCTAATGGG	0.82	94–116
17q21	D17S250^†^	GGAAGAATCAAATAGACAAT	GCTGGCCATATATATATTTAAACC	0.81^§^	151–169
18q21.1	D18S1127*	AGACCCTGGAGAGTGACTGC	TGCCCATGAACTTAGTGTGA	0.86	178–204
18q21.1	D18S1119	CCTATCGTACATGGTGAGTG	CTTGATTTGAACCTAATGACG	0.83	156–170
18q21.1	D18S487	ACAATCAGAAACCCGCCA	AGCTGACTTAGGTAGATTTTCCTCG	0.8	115–127
19q12	D19S875	TGGTTCTGTGATGACTACTACATGC	AACTTGGTTTATGATGTCTCTTGC	0.75	95–123
19q12	D19S414*	CCAGACCTGTCCATCTTGTATGAAT	TTAGAACAACGCTTGGGCATTT	0.77	163–187

*Excluded from the final assays, ^†^dinucleotide markers from the panel used for the analysis of microsatellite instability, ^‡^Het: Heterozygosity according to Dib *et al*.^14^ or ^§^Broman *et al*.^15^.

Markers D17S1353 and D18S1127 were excluded during the optimization step due to weak amplification intensities. The three dinucleotide microsatellite markers D2S123, D5S346 and D17S250 used for the analysis of MSI were also included in the analysis of AI.

For the determination of the individual cut-off values for the definition of AI, DNA from 58 non-tumorous tissues was amplified for a total of 31 microsatellite markers. PCR products were separated and analysed as described in the Supplementary Methods. The range of variation of the amplification of the alleles of each marker using DNA from non-tumorous tissues was determined by dividing the allele ratio (peak area of the shorter allele divided by peak area of the longer allele) of heterozygous markers for each sample with each other as described^10^.

For the determination of AI in the tumours, AI values were calculated as reported^8,9^ by dividing the allele ratios of the normal DNA (N) by the matched tumour DNA (T) and are summarized below.

1 $$AI-value=\frac{peak\,area\,(N,shorter\,allele)}{peak\,area\,(N,longer\,allele)}\times \frac{peak\,area\,(T,\,longer\,allele)}{peak\,area\,(T,\,shorter\,allele)}$$

The frequency of AI at a given microsatellite locus was defined as:

2 $$Frequency\,of\,AI( \% )=\frac{number\,of\,tumours\,with\,AI}{number\,of\,informative\,tumours}\times 100$$

The AI ratio per tumour was defined as:

3 $$AI\,ratio\,per\,tumour=\frac{number\,of\,markers\,with\,AI}{number\,of\,informative\,markers}$$

### Assessment of intra-tumour heterogeneity and limit of detection of multiplex PCR assays

The performance of the CIN classification was analysed in relation to tumour heterogeneity. The intra-tumour variability was assessed of nine tumours each with five areas and the AI ratios of every area were determined. The five areas were selected to represent central parts of the tumour as well as areas located proximal, distal and/or lateral to the centre near the respective tumour margins.

As tumours are usually a mixture of normal and cancer cells, we analysed the limit of detection of our assays by dilution experiments. The initial tumour cell contents of four tumours were determined by a pathologist and DNA from the tumours was mixed with the corresponding normal DNA. The resulting tumour cell contents are included in Supplementary Table S3 and AI ratios were determined for every mixing ratio.

### OncoScan analysis

DNA from 30 GC was analysed for genome wide copy number variations (CNV) using the Affymetrix OncoScan FFPE assay kit, which is based on molecular inversion probe (MIP) technology and is optimized for highly degraded FFPE samples with a probe interrogation site of 40 bp. Samples (80 ng DNA) were processed by IMGM Laboratories GmbH (Martinsried, Germany) according to the protocol of the manufacturer (Affymetrix, Santa Clara, CA). A set of quality metric parameters, normalized log intensity ratios (sample/reference) and B-allele frequencies (BAF) were generated.

Copy number aberrations of the samples were analysed based on the log intensity ratio (log2R) using the SNP-FASST2 algorithm implemented in the Nexus Express Software for OncoScan 3.1 (Biodiscovery, Inc.2014, El Segundo, CA). The algorithm generates segmentation calls based on both the log2R and BAF data. The significance threshold for segmentation was set at 1.0e^−5^, the calling threshold for hemizygous losses at log2R < −0.2 and for single copy gains at >0.2 essentially as described^17^. For the other settings, the default values were used. By default, each sample was centered to the median log2R automatically. Whole genome plots of all samples were visually inspected and manual recentering was performed, if log2R indicated losses or gains but the BAF plot showed a normal three band pattern. As allelic imbalances may represent copy number gains or losses, which in some cases could not be unequivocally identified by the OncoScan assay and which cannot be distinguished per se by the microsatellite assays, we included the calls of AI by the OncoScan assay in all our analysis.

For the purpose of our study we determined the number of altered chromosomal arms per tumour. Essentially in line with the TCGA study a chromosomal arm was considered to be altered if at least 80% of the arm was lost, gained or demonstrated AI^6^. Tumours were classified as CIN-High, according to the TCGA study, if they showed at least one altered chromosomal arm, except for chromosome 18 and 21q^6^. The percentage of alteration per chromosomal arm was calculated by dividing the length of the particular alteration of the p- or q-arm through the total length of the respective p- or q-arm. The ratio of chromosomal alterations per tumour was defined as:

4 $$Ratio\,of\,chromosomal\,alterations\,per\,tumour=\frac{number\,of\,altered\,chromosomal\,arms}{\begin{array}{c}total\,number\,of\,chromosomal\,arms\\ (n=36)\end{array}}$$

### Statistical analysis

The individual cut-offs for the determination of AI were estimated from the lower and upper bounds of the bootstrapped two-sided 95% confidence intervals of the 2.5% and 97.5% quantiles, respectively. Comparison between the results of the microsatellite based multiplex PCR assays and the OncoScan method was performed using the Pearson correlation coefficient. Two-sided Chi-Square Tests were applied to detect associations of AI and CIN with clinical-pathological characteristics of the patients on exploratory 5% significance levels.

For statistical evaluation of the microsatellite based CIN classification in relation to tumour heterogeneity, a crossing probability was calculated from a data set comprising 45 tumour areas from nine patients. The probability that patients would be allocated to a different CIN classification due to intra-tumour variability of AI was computed by the following formula.

5 $$\begin{array}{rcl}{\rm{Crossing}}\,{\rm{probability}}\,{\rm{CP}} & = & {\rm{P}}(\mbox{''}\mathrm{patient}\,{\rm{changes}}\,{\rm{CIN}}\,\mathrm{classification}\mbox{''})\\  & = & {\int }_{-\infty }^{\infty }\,{\rm{P}}(\mbox{''}\mathrm{patient}\,{\rm{with}}\,{\rm{AI}}\,{\rm{ratio}}\\  &  & \times \,{\rm{changes}}\,{\rm{CIN}}\,\mathrm{classification}\mbox{''}){\rm{dx}}\\  & = & {\int }_{-\infty }^{\infty }\,{\rm{P}}(\mbox{''}\mathrm{patient}\,{\rm{changes}}\,{\rm{CIN}}\,\mathrm{classification}\mbox{''}|\mbox{''}\mathrm{AI}\,{\rm{ratio}}\,{\rm{x}}\mbox{''})\\  &  & \cdot \,{\rm{P}}(\mbox{''}\mathrm{AI}\,{\rm{ratio}}\,{\rm{x}}\mbox{''})\,{\rm{dx}}\\  & = & {\int }_{-\infty }^{\infty }\,(1-{\rm{\Phi }}(\frac{|{\rm{x}}-{\rm{c}}|}{{{\rm{s}}}_{{\rm{w}}}/\sqrt{{\rm{r}}}}))\cdot {\rm{\phi }}(\frac{{\rm{x}}-\bar{{\rm{x}}}}{{{\rm{s}}}_{{\rm{b}}}}){\rm{dx}}.\end{array}$$

Here, *φ* and Φ denote the density and cumulative distribution function of the standard normal distribution. The parameters s_w_ and s_b_ refer to the empirical estimates of the standard deviation within repeated measurements (=intra-tumour variability) and between the patients’ AI ratios (=inter-tumour variability). The cut-off value (AI ratio ≧ or < 0.2) of concern is denoted by c. The intra-tumour variability decreases by a multiplicative factor equal to the inverse square root of the number of r (repeated measurements) on a patient’s AI analysis when an average AI ratio is used for risk prediction. Thus, the reliability of a prediction can be increased through the number of measurements made on the AI analysis of a patient. All statistical analyses were performed using IBM SPSS Statistics, Version 24 (IBM Corp., Armonk, NY) and R version 3.1.0 (R Foundation for Statistical Computing, Vienna, Austria).

## Results

**Figure 1:**
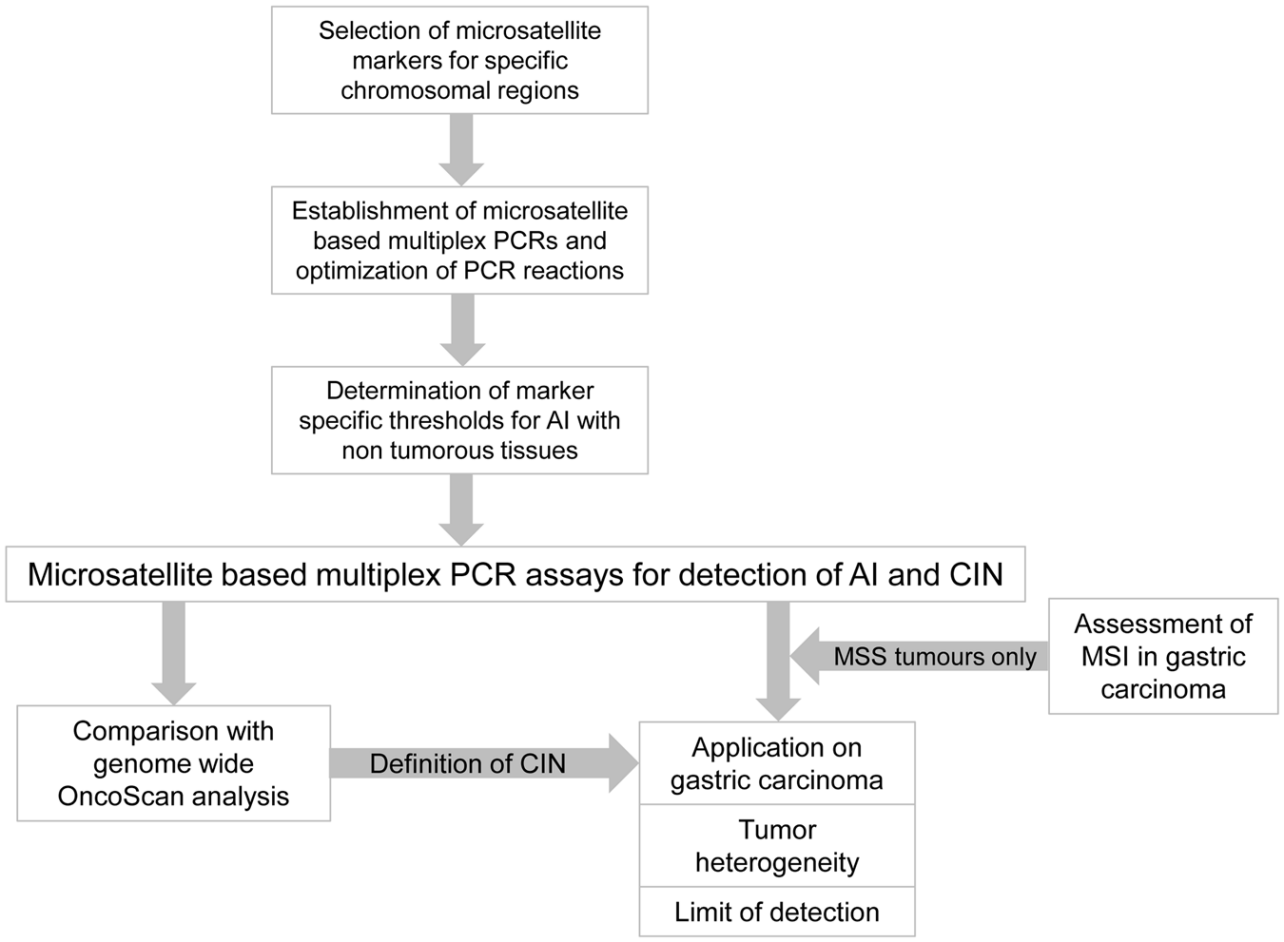
Study design of the establishment of the microsatellite based multiplex PRC assays for the detection of allelic imbalance (AI) and chromosomal instability (CIN). A two-step protocol with the determination of MSI first was used and only microsatellite stable (MSS) gastric carcinomas were evaluated for AI and CIN. MSI, microsatellite instability.

An overview of our study design is shown in Fig. 1. In brief, microsatellite loci were selected and multiplex PCR reactions were designed and optimized. Individual threshold values for each marker for the determination of AI were defined by analysing non-tumorous tissues. Performance of the assay was tested on a cohort of GC and the results were compared to those from the Affymetrix OncoScan assay in a subset of the tumours to define a classification of CIN based on both methods. Performance of the multiplex PCR assays was in addition analysed in relation to tumour heterogeneity and the limit of detection of CIN with respect of the amount of tumour cells was determined.

### Determination of individual cut-off values for the definition of AI and establishment of the final multiplex assays

Individually determined upper and lower threshold values of the variation of the allelic ratios of each microsatellite marker were used to define an AI. Analysis of 58 non-tumorous DNA samples from 11 patients demonstrated specific AI-threshold values of the respective markers in the range from 0.65–1.59 (Table 2). The nine markers D8S1801, D17S946, D6S1713, D19S414, D5S2107, D4S1534, D16S3125, D8S261 and D17S1832 were excluded from further analysis due to weak amplification efficiencies, low heterozygosity rates or the occurrence of various stutter bands which complicated the evaluation of the allele ratios.

**Table 2:** Upper and lower threshold values for the definition of AI of the microsatellite markers (n = 22) in the final multiplex assays

Chromosomal regions	Microsatellite marker	Number of non-tumorous tissues	Lower threshold value	Upper threshold value
2p21	D2S123	62	<0.82	>1.29
4q22	D4S423	126	<0.77	>1.43
5q11.2	D5S624	69	<0.67	>1.34
5q21	D5S346	95	<0.67	>1.18
6p25	D6S1617	93	<0.81	>1.33
7q21	D7S492	99	<0.68	>1.26
7q31	D7S486	36	<0.65	>1.56
8p23.1	D8S552	86	<0.66	>1.24
8q24.21	D8S1793	126	<0.71	>1.43
8q24.21	D8S1720	116	<0.73	>1.34
9p21	D9S157	36	<0.88	>1.16
9p21	D9S171	52	<0.83	>1.18
12p12	D12S1682	98	<0.80	>1.17
12p12	D12S1631	87	<0.77	>1.26
16q23	D16S507	74	<0.67	>1.39
17p13	D17S796	95	<0.69	>1.30
17q12	D17S1861	120	<0.78	>1.30
17q12	D17S1872	107	<0.80	>1.37
17q21	D17S250	93	<0.64	>1.45
18q21	D18S487	126	<0.81	>1.20
18q21	D18S1119	49	<0.74	>1.38
19q12	D19S875	83	<0.75	>1.49

AI, allelic imbalance.

The remaining 19 microsatellite markers covering 14 chromosomal regions were finally combined in four multiplex reactions. The composition of the final multiplex assays and the covered chromosomal regions are listed in Table 3.

**Table 3:** Composition of the four multiplex PCR reactions of the final microsatellite assays.

	Chromosomal regions	Microsatellite marker	Fluorescence dyes*	Primer concentration [µM]
Multiplex PCR 1	12p12^†^	D12S1682	HEX	4
	16q23	D16S507	HEX	6
	8q24^†^	D8S1793	FAM	4
	9p21^†^	D9S171	FAM	1
Multiplex PCR 2	19q12	D19S875	HEX	2
	17p13	D17S796	HEX	2
	4q22	D4S423	FAM	2
	9p21^†^	D9S157	FAM	2
	18q21^†^	D18S1119	FAM	8
Multiplex PCR 3	18q21^†^	D18S487	HEX	2
	5q11	D5S624	HEX	2
	17q12^†^	D17S1861	FAM	2
	8q24^†^	D8S1720	FAM	5
	8p23	D8S552	FAM	2
	7q31	D7S486	ATTO550	2
Multiplex PCR 4	17q12^†^	D17S1872	HEX	4
	6p25	D6S1617	FAM	3
	7q21	D7S492	FAM	1
	12p12^†^	D12S1631	FAM	4

*Forward primers were labeled at 5′- end, ^†^regions are covered with two markers.

### Analysis of MSI and AI in gastric carcinomas

**Figure 2:**
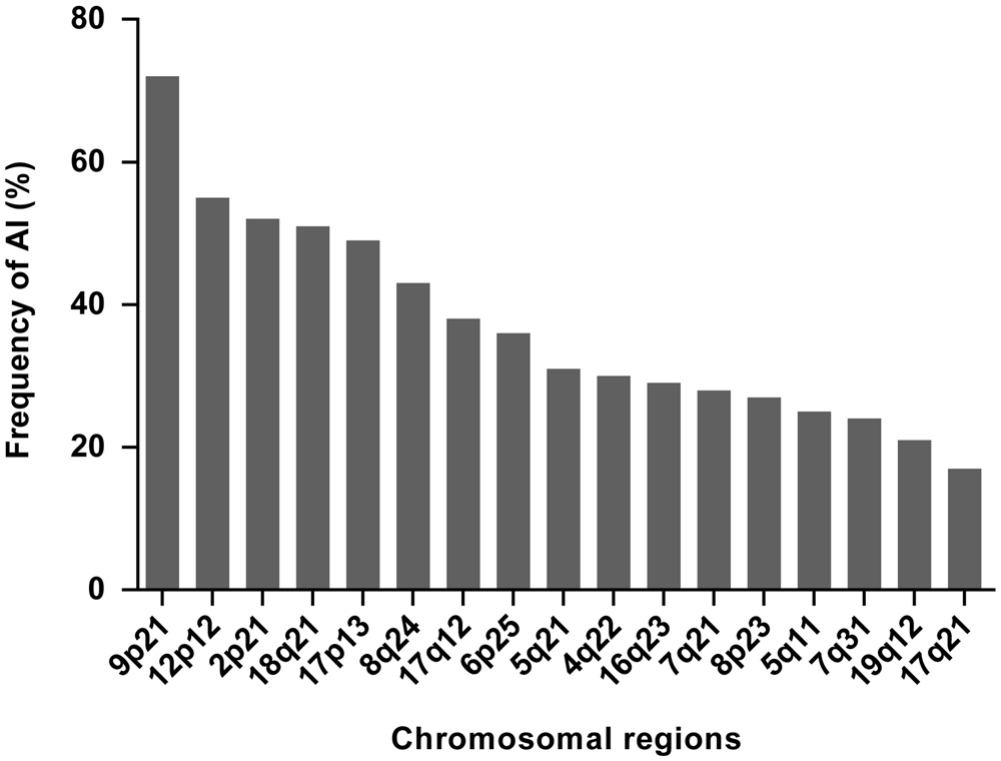
Frequency of AI at 17 chromosomal regions. The bars represent the percentage of tumours with AI per number of informative markers. The chromosomal regions 8q24, 9p21, 12p12, 18q21 and 17q12 are covered with two markers and AI was counted when at least one of the both markers detected AI. AI, allelic imbalance.

All tumours were first analysed for MSI, and 10 of the 100 GC (10%) were MSI and 90 (90%) were MSS. Performance of the multiplex PCR assay was tested only in the MSS tumours. AI at 9p21, 12p12, 2p21 and 18q21 was found in 71%, 55%, 53% and 53% of the tumours and represented the most frequent alterations. AI at 17q21 and 19q12 were with 18% and 22% the less frequent alterations. Results are summarized in Fig. 2 and in Supplementary Table S4.

### Comparison of the microsatellite multiplex PCR assays with the OncoScan platform

A subset of 30 gastric tumours was analysed with the OncoScan assay which enables a genome-wide analysis of copy number gains or losses and in addition, indicates regions of AI.

**Figure 3:**
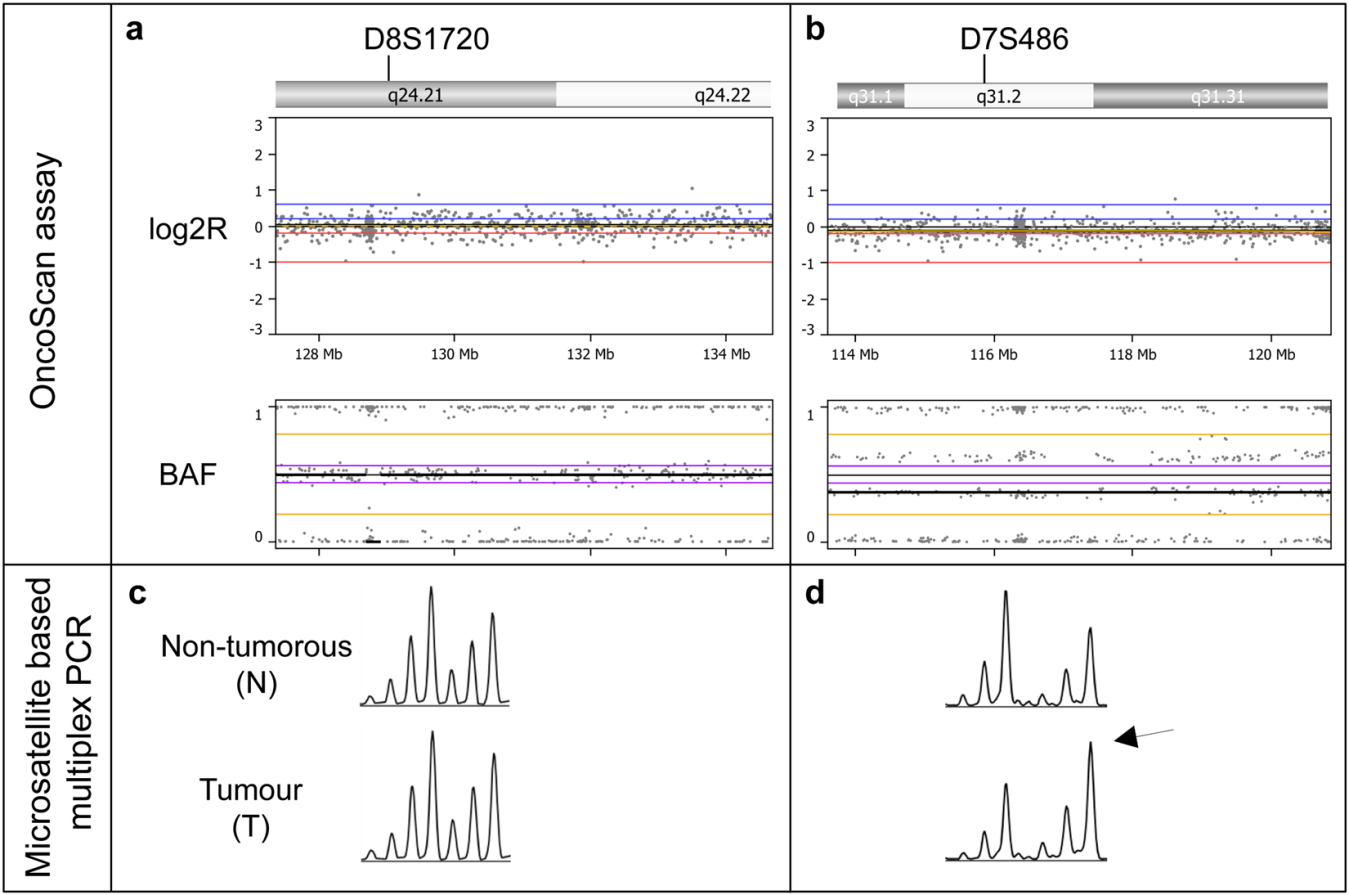
Examples of tumours, which are negative **(a,c)** and positive for AI **(b,d)** in the OncoScan **(a,b)** and the microsatellite based multiplex PCR assays **(c,d)**. The Nexus Express software displays logR and BAF graphs for every chromosomal arm. In the log2R plot each dot represents copy number values which are calculated from signal intensities of a tumour sample compared to a normal reference and the B-allele frequency generates allelic information at each SNP position. At the region of the microsatellite marker D8S1720 **(a)** the tumour has zero values in the log2R and a normal three-band pattern in the BAF plot, which indicates no AI. At the region of the microsatellite marker D7S486 **(b)** the BAF plots show a clear four band pattern, which indicates AI. The relation of the allele intensity of the first and second allele of the respective microsatellite locus is not shifted at the microsatellite marker D8S1720, indicating no AI **(c)**. At the marker D7S486 **(d)** a clear shift is shown in the allele intensities in the tumour (arrow) in comparison to the non-tumorous tissue (N), which indicates AI. AI, allelic imbalance; BAF, B-allele frequency; log2R, log intensity ratio.

**Figure 4:**
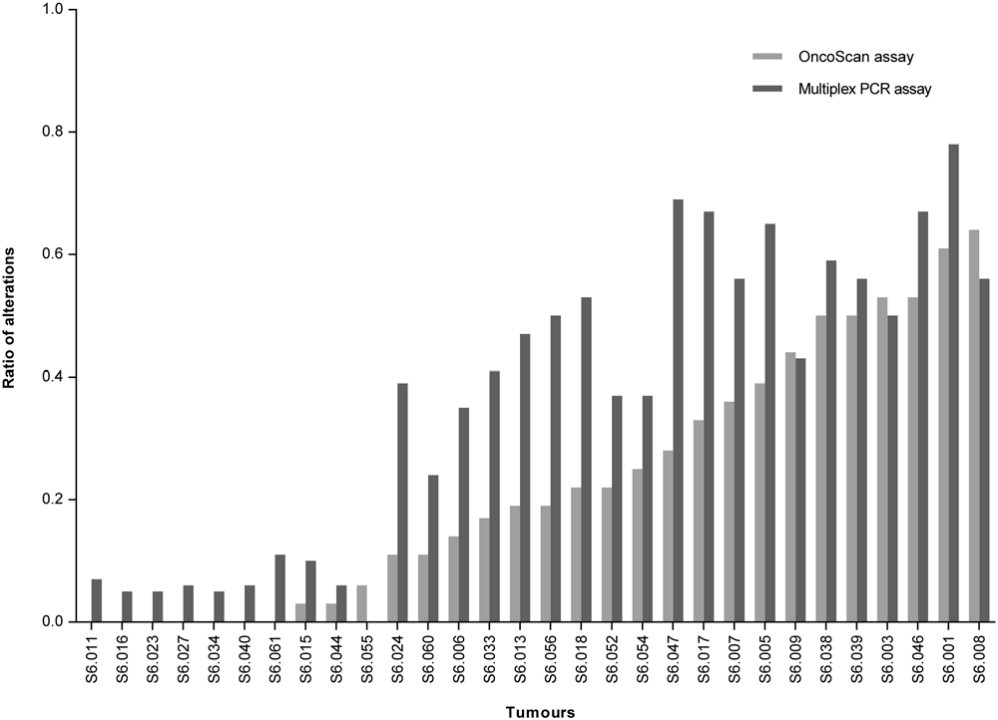
Overview of the ratios of alterations in the 30 tumours detected with the OncoScan assay compared to the microsatellite based multiplex PCR assays.

**Figure 5:**
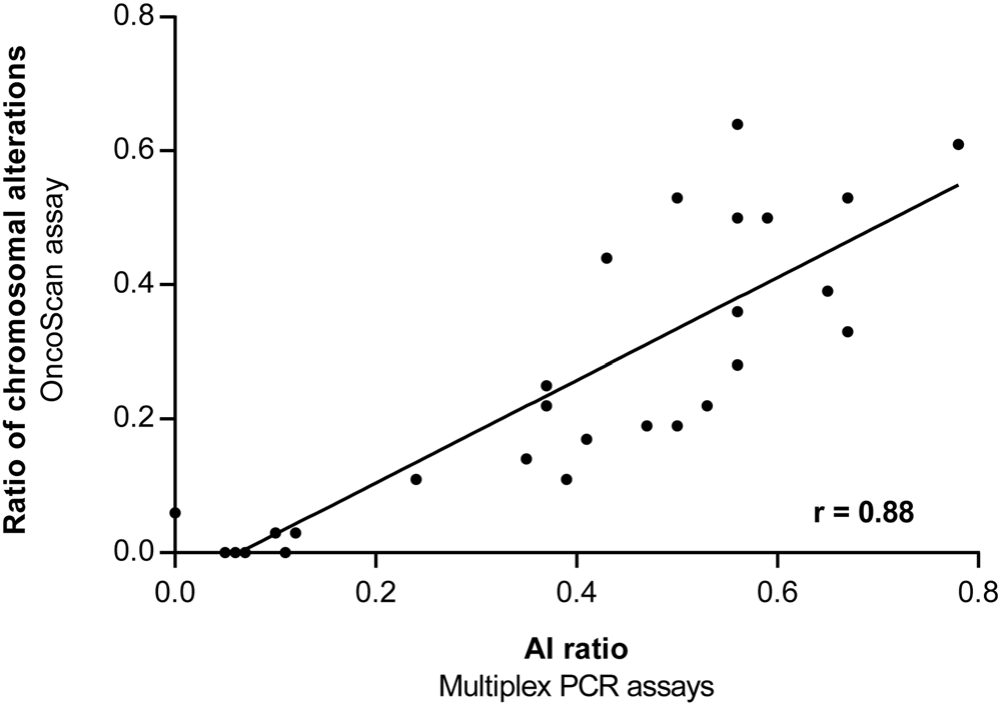
Correlation of the ratios of chromosomal alterations detected with the OncoScan assay compared to the AI ratios determined with the microsatellite based multiplex PCR assays. AI, allelic imbalance.

We compared the occurrence of chromosomal alterations determined by the OncoScan analysis with the results of AI at the respective microsatellite locus determined by multiplex PCR and a concordance of 84% was observed. Examples of AI detected with the OncoScan assay compared to AI detected with the microsatellite multiplex assays are shown in Fig. 3. Next we determined the genome wide extent of alterations affecting all chromosomal arms. According to TCGA data^6^, a chromosomal arm was considered to be altered if at least 80% of one arm was altered. An overview of the ratios of chromosomal alterations in the 30 analysed tumours detected by the OncoScan assay in comparison with the AI ratios per tumour determined by the microsatellite based multiplex PCR assays is shown in Fig. 4 and Supplementary Table S5. A strong correlation with a correlation coefficient of 0.88 was found for the ratios of alterations detected by the two methods (Fig. 5).

### Determination of CIN according to definition of TCGA based on OncoScan and microsatellite analysis

We classified the 30 tumours analysed with the OncoScan assay as having high chromosomal instability (CIN-H) essentially according to the definition of TCGA^6^. Accordingly, 23 (77%) of the 30 tumours were CIN-H and 7 (23%) were CIN-Low (-L) tumours. Taken this OncoScan based CIN classification as standard, we searched for a corresponding threshold value of the AI ratio determined with the microsatellite assay. A high concordance of 90% for the CIN classification of both methods was observed for two possible cut-off values. A cut-off value of an AI ratio of <0.11 resulted in the false positive classification of 10% of the cases as CIN-H, whereas a cut-off of ≤0.24 resulted in the false positive classification of 10% of the cases as CIN-L.

Considering the AI ratios of the 90 gastric carcinomas analysed with the microsatellite assay, values in the range from 0 to 0.78 were observed with 15 tumours demonstrating rates between 0.12 and 0.22. The median AI ratio of these 15 tumours was 0.2 and we propose a final cut-off value of ≧0.2 for the classification of tumours as CIN-H and <0.2 as CIN-L.

According to this classification 20 of the 90 gastric carcinomas (22%) were CIN-L and 70 (78%) were CIN-H. Correlation of CIN with clinical-pathological characteristics of the patients demonstrated that CIN-H occurred more frequently in intestinal type tumours (P < 0.001), in tumours with a lower tumour grade (P = 0.036) and with proximal tumour location (P = 0.009) (Supplementary Table S6).

### Microsatellite based CIN classification and tumour heterogeneity

We analysed the robustness of our established microsatellite based CIN classification in relation to tumour heterogeneity. Seven of the nine analysed tumours demonstrated a concordant classification in all five areas, one tumour in four areas and one tumour in three areas (Supplementary Fig. S1). The probability that tumours would be allocated to a different CIN classification due to intra-tumour variability of the AI ratio was calculated. For a single measurement (r = 1) the crossing probability was 10.3%. Assuming an increased number of analysed tumour areas per patient, the resulting crossing probability showed that an average AI ratio reduces the crossing probability to 7.4% and 6.1% for the analysis of two and three tumour areas.

### Limit of detection of CIN by the microsatellite based multiplex PCR assays

DNA from four tumours was diluted with corresponding normal DNA and the AI ratios were determined. The initial tumour cell contents determined by a pathologist were 60%, 70% (for two tumours) and 90%. A stable classification in CIN-H was given at tumour cell contents between 24%-35%. Mixing ratios and results are included in Supplementary Table S3.

## Discussion

Chromosomal unstable gastric and oesophageal adenocarcinomas have recently described as one of the four molecular subgroups identified in comprehensive “omics” based studies by the respective TCGA consortia^3,6^, but knowledge of the clinical significance of these molecular classes is still limited. In particular, for the determination of CIN an adequate, cost efficient diagnostic tool, which can be used for the analysis of FFPE tissues in a high number of cases, is needed.

In this study, we describe the successful establishment of a microsatellite based multiplex PCR assay to detect AI as a surrogate for CIN. As a reliable determination of AI is only possible in microsatellite stable (MSS) tumours, MSI was assessed in a preselection step and only MSS tumours were further evaluated for CIN. We emphasize that the term CIN is used according to the TCGA studies and refers to a static measurement of the amount of copy number variations and allelic imbalances observed in a tumour.

Microsatellite analysis allows the detection of AI by comparing the intensities of the paternal and maternal alleles between normal and tumour tissues of heterozygous patients and then by calculating a quotient of the respective allele ratios between normal and tumour tissue. A standardized cut-off value for the determination of AI is not clearly defined and various studies used cut-off values in the range below 0.5–0.6 or above 1.5–2.0, which correspond to a reduction in the intensity of one of the alleles of at least 40–50%^8,9,18–21^. For our assay, we experimentally determined individual cut-off values for each microsatellite marker. This approach has been described in only a few studies^10,11^ and takes into account marker specific amplification characteristics and finally allows a more sensitive detection of AI. The comparison of the results regarding the AI ratios determined by our PCR assays with the ratio of genome wide chromosomal alterations detected by the OncoScan assay, revealed a high correlation (r = 0.88). This indicates that in good approximation the PCR assays reliably and specifically reflects the extent of chromosomal alterations occurring on a genome wide level. Some of the discrepancies between the two methods were related to the occurrence of balanced gains detected in the OncoScan assay, which are not detectable by microsatellite analysis or to AI values very close to the cut-off value of the respective microsatellite marker. Some crucial advantages of our assay are that it is easy to perform, is cost-efficient, and is applicable for DNA isolated from FFPE tissues. Thus, it could be routinely applied in a clinical diagnostic setting or used in large translational studies. In addition, we addressed the sensitivity of the microsatellite based assay and demonstrated that the assay can be reliably used if the tumour cell content is in a range of 24%-35%. Furthermore, we demonstrated a rather stable performance of CIN classification based on this assay in relation to tumour heterogeneity.

Other methods, as for example fluorescence *in situ* hybridization (FISH), SNP arrays or comparative genomic hybridization (CGH) analysis, have been used for the determination of CIN^2^. However, FISH is highly labour intensive and usually restricted to the analysis of few chromosomal regions, whereas SNP arrays and CGH provide information about chromosomal alteration on a genome wide level, but are rather expensive and are mainly used for the analysis of DNA from fresh frozen tumours. Furthermore the OncoScan assay, which we used in our study, also requires special equipment.

In our study 78% of the tumours were CIN-H. This is essentially in line with a frequency of 79% CIN-H tumours described recently for microsatellite stable tumours located in the proximal stomach and oesophagus^7^. In addition the CIN-H phenotype was associated with the intestinal tumour type and with proximal tumour location, which is similar to previous reports^3,6,7,9,22^. The finding of an association of CIN-L with higher tumour grade, most likely reflects the association of CIN-L with non-intestinal type tumours, which usually are poorly differentiated neoplasms.

Our established microsatellite assay also has some limitations. The assay is based on a comparison of the amplification of the respective microsatellite alleles between non-tumorous and tumour tissues and thus requires an additional DNA extraction from non-tumorous tissue. Furthermore, microsatellite analysis indicates AI and does not allow the clear distinction between gain or loss of a chromosomal region. This however, is not essentially required for the more global classification of chromosomal instability which was the purpose of our study.

Furthermore, the definition of a specific threshold to define CIN-H and CIN-L tumours has to be critically considered. Chromosomal unstable gastric tumours have been broadly defined as tumours having extensive somatic copy number aberrations by the TCGA network^3^. A more specific definition of CIN was used for the genomic characterization of oesophageal and gastric adenocarcinomas published recently^6^. Regarding microsatellite analysis there is no standardized threshold to classify tumours as CIN-H or -L. Taking the classification of CIN by the TCGA as a guideline^6^ we defined a corresponding threshold value of the AI ratio of ≧0.2 for the classification of tumours as CIN-H and <0.2 as CIN-L.

Watanabe *et al*. (2012) classified colorectal carcinoma as CIN-H or CIN-L when the LOH ratio (AI ratio) was ≥33% or <33% analysing seven microsatellite markers of five chromosomal regions^19^. In this study CIN-H tumours were further subclassified as a mild or severe type when the LOH ratio was <75% or ≥75%. A similar LOH based classification of CIN in three groups demonstrated a significant association with the assessment of CIN determined by the DNA index using image cytometry and this three-part CIN classification was shown to have a strong prognostic relevance^20^. These findings underline the appropriateness of microsatellite analysis for the determination of CIN but also indicate that besides the classification into two groups a more differentiated graduation of CIN might be more appropriate to detect biological differences of the tumours.

Considering the frequencies of altered chromosomal regions in our study showed that AI at 9p21, 2p21 and 17p13 was among the most frequent alterations. In various studies loss at 9p21 and 17p13 have been reported in GC in a broad range from 11–57% and 33–71%^9,22–24^. Our results of an AI of 72% at 9p21 and of 49% at 17p13 are within this range and the overall differences may be related to the different techniques used or to specific characteristics of the analysed patient cohorts. Comparing the detected AI ratios by the microsatellite based assays with the ratios of chromosomal alterations detected by the OncoScan assay revealed that in general our assay showed higher ratios of AI. This higher sensitivity may be related to our individual definition of specific cut-off values for each marker. In addition, the fact that we mainly used markers of chromosomal regions which are specifically altered in GC may explain the higher AI ratios^3,6,9,22^.

In conclusion, we describe the successful establishment of a microsatellite based PCR method to detect AI as a surrogate for CIN and its application on GC using a two-step protocol with the assessment of MSI first and determination of CIN only in microsatellite stable tumours. The method is easy to perform, allows a cost effective analysis of large GC cohorts and thus could substantially contribute to a better characterization and understanding of the biological and clinical relevance of the recently identified molecular subgroups of these tumours.

## Electronic supplementary material

Supplementary Information

## Data Availability

Data are available from the corresponding author upon reasonable request.
