# Supplementary material for: A microsatellite based multiplex PCR method for the detection of chromosomal instability in gastric cancer
Source: Sci Rep. 2018 Aug 22;8:12551. doi: 10.1038/s41598-018-30971-z (PMC6105665; doi:10.1038/s41598-018-30971-z)
Supplement: Supplementary file 1 — Supplementary Information [file 41598_2018_30971_MOESM1_ESM.pdf]

Supplementary Information

A microsatellite based multiplex PCR method for the detection of chromosomal instability in gastric cancer

Meike Kohlruss, Magdalena Reiche, Moritz Jesinghaus, Bianca Grosser, Julia Slotta-Huspenina, Alexander Hapfelmeier, Lukas Bauer, Alexander Novotny, Wilko Weichert, Gisela Keller

Contents

Supplementary Methods

Supplementary Reference

Supplementary Tables S1 – S6

Supplementary Figure S1

Supplementary Methods

Analysis for microsatellite instability (MSI)

MSI was analysed using the five markers BAT25, BAT26, D2S123, D5S346 and D17S250 recommended by the National Cancer Institute[1]. A multiplex PCR with fluorescence-tagged primers was performed using the Type-it Microsatellite PCR kit (Qiagen, Hilden, Germany) on non-tumorous and tumour DNA.

Cycle conditions of microsatellite based multiplex PCR

Cycle conditions were as follows: after an initial step of 95°C for 5 min, 32 cycles were performed consisting of denaturation at 95°C for 30 sec, annealing at 58°C for 90 sec and extension at 72°C for 30 sec and final extension at 60°C for 30 min.

Fragment analysis of PCR products

Separation and detection of the PCR products was performed in a 3130 Genetic Analyzer (Applied Biosystems, Foster City, CA) loaded with POP-7 polymer (Applied Biosystem) and using ROX-500 Genescan (Thermo Scientific) as size standard. Samples were analysed with the GeneMapper Software 5 (Applied Biosystem).

Supplementary Reference

1. Boland, C. R. et al. A National Cancer Institute Workshop on Microsatellite Instability for cancer detection and familial predisposition: development of international criteria for the determination of microsatellite instability in colorectal cancer. *Cancer Res.* **58,** 5248-5257 (1998).

Supplementary Table S1 Characteristics of the gastric cancer patients

**Category**	**Value**	**n**	**%**
Cases	Total	100	100
Age	Median	71	
	Range	37.9 - 90.9	
Sex	Male	62	62
	Female	38	38
Localization	Proximal	22	22
	Middle	33	33
	Distal	34	34
	Total/linitis	7	7
	NA	4	4
Laurén histological subtype	Intestinal	50	50
	Non intestinal	50	50
Tumour grade	G1/2	24	24
	G3/4	76	76
Metastasis Stage	No	81	81
	Yes	19	19
pT *	pT1	24	24
	pT2	16	16
	pT3	24	24
	pT4	36	36
pN *	Negative	45	45
	Positive	55	55
MSI Status	MSI	10	10
	MSS	90	90
CIN Status	CIN-Low	20	22
	CIN-High	70	78

* pT, pN classification according to UICC 2007, MSS, microsatellite stable; MSI, microsatellite instability; CIN, chromosomal instability; NA, not available.

Supplementary Table S2 Composition of the five multiplex PCR reactions for the determination of the individual cut-off values for the definition of allelic imbalance (AI)

	Microsatellite marker	Fluorescence labelling *	Primer concentration [µM]
Multiplex PCR 1	D18S487	HEX	0.5
	D17S796	HEX	2
	D17S1832	FAM	2
	D8S1793	FAM	2
	D8S1801	FAM	2
Multiplex PCR 2	D17S946	ATO	2
	D17S1872	HEX	2
	D17S1861	FAM	0.5
	D8S1720	FAM	2
	D18S1119	FAM	4
Multiplex PCR 3	D19S875	HEX	2
	D5S624	HEX	4
	D16S507	HEX	10
	D4S423	FAM	1.5
	D8S261	FAM	2
	D4S1534	FAM	3
	D8S552	FAM	6
Multiplex PCR 4	D6S1713	HEX	3
	D9S157	FAM	2
	D9S171	FAM	1.5
	D16S3125	FAM	6
	D7S486	ATO	2
	D19S414	ATO	4
Multiplex PCR 5	D6S1617	FAM	2
	D12S1682	HEX	4
	D5S2107	HEX	8
	D7S492	FAM	1
	D12S1631	FAM	3

* Forward primers were labelled at 5’- end

Supplementary Table S3 Results of dilution series

Sample 1			Sample 2		
tumor cell content	AI ratio	CIN classification	tumor cell content	AI ratio	CIN classification
**70%**	0.54	High	**60%**	0.47	High
60%	0.40	High	51%	0.55	High
56%	0.47	High	48%	0.41	High
50%	0.40	High	43%	0.41	High
46%	0.40	High	40%	0.35	High
42%	0.40	High	36%	0.47	High
35%	0.20	Low	30%	0.35	High
28%	0.07	Low	24%	0.24	High
23%	0.07	Low	20%	0.12	Low
20%	0.07	Low	17%	0.18	Low
14%	0.00	Low	12%	0.12	Low
10%	0.00	Low	9%	0.06	Low
Sample 3			Sample 4		
tumor cell content	AI ratio	CIN classification	tumor cell content	AI ratio	CIN classification
**70%**	0.87	High	**90%**	0.87	High
60%	0.78	High	77%	0.87	High
56%	0.60	High	72%	0.67	High
50%	0.67	High	64%	0.80	High
46%	0.80	High	60%	0.73	High
42%	0.73	High	54%	0.87	High
35%	0.60	High	45%	0.67	High
28%	0.47	High	36%	0.33	High
23%	0.53	High	30%	0.33	High
20%	0.40	High	26%	0.27	High
14%	0.40	High	18%	0.40	High
10%	0.47	High	13%	0.33	High

Initial tumor cell contents determined by a pathologist in bold, AI, allelic imbalance; CIN, chromosomal instability.

Supplementary Table S4 Frequency of AI at 17 chromosomal regions

Chromosomal regions	Number of tumors with AI	Number of informative tumors	Frequency of AI (%)
9p21 *	62	87	71
12p12 *	49	89	55
2p21	29	55	53
18q21 *	41	78	53
17p13	33	66	50
8q24 *	38	88	43
6p25	29	76	38
17q12 *	34	90	38
5q21	26	80	33
16q23	23	73	32
4q22	25	80	31
7q21	21	72	29
8p23	19	66	29
7q31	17	68	25
5q11	18	74	24
19q12	14	65	22
17q21	13	73	18

* Covered with two markers: AI was counted when at least one of the both markers detected AI.

AI, allelic imbalance.

Supplementary Table S5 Ratios of chromosomal alterations and AI ratios of the 30 tumors analyzed with OncoScan and the microsatellite based multiplex assays

	OncoScan assay		Microsatellite based multiplex assays		
Tumors	Number of chromosomal arms with alteration	Ratios of chromosomal alterations	Number of markers with AI	Total number of informative markers	AI ratios
S6.008	23	0.64	9	16	0.56
S6.001	22	0.61	14	18	0.78
S6.003	19	0.53	10	20	0.5
S6.046	19	0.53	8	12	0.67
S6.038	18	0.5	13	22	0.59
S6.039	18	0.5	10	18	0.56
S6.009	16	0.44	6	14	0.43
S6.005	14	0.39	11	17	0.65
S6.007	13	0.36	9	16	0.56
S6.017	12	0.33	12	18	0.67
S6.047	10	0.28	11	16	0.69
S6.054	9	0.25	7	19	0.37
S6.018	8	0.22	10	19	0.53
S6.052	8	0.22	7	19	0.37
S6.013	7	0.19	7	15	0.47
S6.056	7	0.19	9	18	0.5
S6.033	6	0.17	7	17	0.41
S6.006	5	0.14	6	17	0.35
S6.024	4	0.11	7	18	0.39
S6.060	4	0.11	4	17	0.24
S6.055	2	0.06	0	15	0
S6.015	1	0.03	2	20	0.1
S6.044	1	0.03	1	17	0.06
S6.011	0	0	1	15	0.07
S6.016	0	0	1	20	0.05
S6.023	0	0	1	21	0.05
S6.027	0	0	1	18	0.06
S6.034	0	0	1	20	0.05
S6.040	0	0	1	17	0.06
S6.061	0	0	2	18	0.11

AI, allelic imbalance.

Supplementary Table S6 Association of CIN with Laurén histological subtype and clinical pathological characteristics

		CIN-High	CIN-Low	p-value†
Laurén histological subtype	intestinal	40	1	
	non intestinal	30	19	**0.000**
pT *	1/2	30	7	
	3/4	40	13	0.529
pN *	negative	32	8	
	positive	38	12	0.650
Tumour grade	1/2	19	1	
	3/4	51	19	**0.036**
Localization	distal/middle/total	48	20	
	proximal	18	0	**0.009**

* pT, pN classification according to UICC 2007, † two-sided Chi-Square Test, p-values < 0.05 in bold.
CIN, chromosomal instability.

Supplementary Figure S1

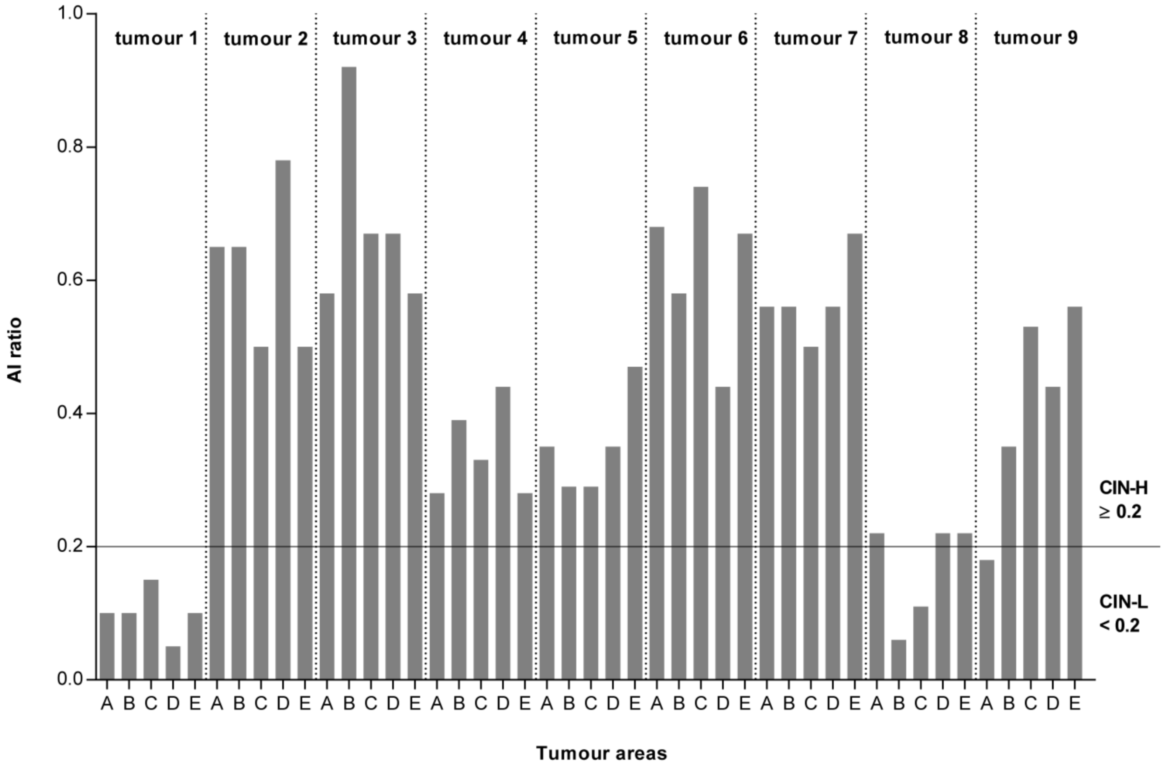

Supplementary Figure S1 Microsatellite based CIN classification and tumour heterogeneity

AI ratios were calculated for nine tumours each with five tumour areas (A-E).

CIN-L, low chromosomal instability; CIN-H, high chromosomal instability; AI, allelic imbalance.
